# Determination of Chemical Composition, In Vitro and In Silico Evaluation of Essential Oil from Leaves of *Apium graveolens* Grown in Saudi Arabia

**DOI:** 10.3390/molecules26237372

**Published:** 2021-12-04

**Authors:** Ahmed I. Foudah, Mohammed H. Alqarni, Aftab Alam, Mohammad Ayman Salkini, Pravej Alam, Faisal K. Alkholifi, Hasan S. Yusufoglu

**Affiliations:** 1 Department of Pharmacognosy, College of Pharmacy, Prince Sattam Bin Abdulaziz University, Al-Kharj 11942, Saudi Arabia; a.foudah@psau.edu.sa (A.I.F.); m.alqarni@psau.edu.sa (M.H.A.); m.salkini@psau.edu.sa (M.A.S.); 2 Department of Biology, College of Science and Humanities, Prince Sattam Bin Abdulaziz University, Al-Kharj 11942, Saudi Arabia; alamprez@gmail.com; 3 Department of Pharmacology, College of Pharmacy, Prince Sattam Bin Abdulaziz University, Al-Kharj 11942, Saudi Arabia; f.alkholifi@psau.edu.sa; 4 Department of Pharmacognosy & Pharmaceutical Chemistry, College of Dentistry & Pharmacy, Buraydah Private Colleges, Buraydah 81418, Saudi Arabia

**Keywords:** *Apium graveolens*, essential oil, anti-inflammatory, antimicrobial, antioxidant, isocnidilide, molecular docking

## Abstract

The aim of this study was to explore the composition and evaluate the in silico and in vitro antioxidants and antimicrobial and anti-inflammatory effects of *Apium graveolens* var. dulce leaves essential oil (AGO) collected from Al-Kharj (Saudi Arabia). AGO was isolated using the hydro-distillation method, and its composition was studied using gas-chromatography-mass Spectrometry (GC–MS), antimicrobial activities using well diffusion assay, and antioxidant and anti-inflammatory activities using spectrophotometric methods. The pharmacological activities of their major compounds were predicted using PASS (prediction of activity spectra for substances) and drug-likening properties by ADME (absorption, distribution, metabolism, and excretion) through web-based online tools. Isocnidilide (40.1%) was identified as the major constituent of AGO along with β-Selinene, Senkyunolide A, Phytyl acetate, and 3-Butylphthalide. AGO exhibited a superior antibacterial activity, and the strongest activity was detected against Gram-positive bacteria and *Candida albicans.* Additionally, it exhibited a weaker antioxidant potential and stronger anti-inflammatory effects. PASS prediction supported the pharmacological finding, whereas ADMET revealed the safety of AGO. The molecular docking of isocnidilide was carried out for antibacterial (DNA gyrase), antioxidant (tyrosinase), and anti-inflammatory (cyclooxygenase-2) activities. The docking simulation results were involved hydrophilic interactions and demonstrated high binding affinity of isocnidilide for anti-inflammatory protein (cycloxygenase-2). The presence of isocnidilide makes AGO a potential anti-inflammatory and antimicrobial agent. AGO, and its major metabolite isocnidilide, may be a suitable candidate for the future drug development.

## 1. Introduction

*Apium graveolens* (*A. graveolens*) is a small-sized annual or biennial herb that is a member of the Apiaceae family. It is extensively cultivated as a garden crop throughout Europe, Asia, Africa, and certain areas of South and North America. Two varieties of this plant exist, namely *A. graveolens* var. dulce, commonly called ‘celery’, and *A. graveolens* var. rapaceum, commonly called ‘celeriac’ [1]. Three kinds of celery are cultivated for culinary use: celery roots (*A. graveolens* var. rapaceum), celery stalks (*A. graveolens* var. dulce), and leaf celery (*A. graveolens* var. secalinum), the latter one being characterized by a more assertive flavor than the other two varieties [2].

Celery is a well-known fragrant herb and spice that is widely grown in central area of Saudi Arabia. Raw, cooked, or processed celery is used in soups, seasonings, convenience foods such as sausages, sauces, and salads, as well as in salad dressings and salad dressings [3]. Traditionally, the whole plant is consumed for the treatment of arthritis, rheumatic pain [4], jaundice, female obstruction, and genitourinary treatments [5]. *A. graveolens* has been used in traditional medicine to treat toothaches, diarrhea, hypertension, bronchopulmonary, liver, and asthma illness, and as a diuretic for bladder and kidney complaint [6,7].

A variety of essential oils (terpenes, phthalides, and aldehydes) contribute to the distinctive odor and flavor of celery [8]. Celery contains a variety of phytochemicals, the most important of which are limonene, phthalides, β-salinene, coumarins, spathulenol, and flavonoids (apiin), which are the primary constituents of essential oils [9]. It has a fresh and strong smell note due to the high concentration of limonene, both the enantiomer (R)-(+)-limonene with a citrusy odor and the enantiomer (S)-(−)-limonene with a pine-fresh odour [10]. As a result, celery seed essential oil is frequently utilized in the cosmetics sector, particularly in perfumery, as a fragrance component.

The antioxidant and non-toxic properties of essential oil of *A. graveolens* have been well established by many investigators [11,12,13,14]. Recently, Yildiz et al. evaluated the antioxidant compounds present in *A. graveolens* leaves oil, using different antioxidant methods, and results showed good antioxidant effects [15]. Similarly, Nagella et al., reported that the essential oil of *A. graveolens* had good antioxidant potential against 2,2-Diphenyl-1-picrylhydrazyl (DPPH)-induced free radical [9]. The essential oil and solvent extracts of *A. graveolens* were reported to exhibit cardioprotective [7], hepatoprotective [16], and nephroprotective properties [17]. In general, the chemical composition of volatile oils varies significantly, depending on a variety of factors, including geographical, soil, and climate conditions, and there have been various studies that have demonstrated the variation in essential oils in Apiaceae species in recent years [18,19]. Swollen joints, redness, joint pain or stiffness, and loss of joint functions are the major symptoms of inflammation, and it is closely associated with an increased production of ROS (reactive oxygen species) and RNOS (reactive nitrogen oxide species) [20]. Nonsteroidal anti-inflammatory drugs (NSAIDs) such as ibuprofen, diclofenac, aspirin, and naproxen are anti-inflammatory drugs currently used for inflammation treatments, and these anti-inflammatory agents are known to cause severe side-effects [21].

*A. graveolens* is widely available in the Al-Kharj region of central Saudi Arabia, where it is abundantly farmed and sold in malls, supermarkets, and local markets. Despite various international research, the cultivation of celery leaves in Saudi Arabia (Al-Kharj area) has not yet been investigated for ethnopharmacological effects. It will be helpful to preserve and promote the value of traditional remedies and indigenous knowledge.

In this perspective, the aim of the present study was to investigate the volatile chemical compounds and the biological properties of the essential oil, such as in vitro antimicrobial, antioxidant, and anti-inflammatory properties, were investigated. In addition, to account for and support the in vitro activity data, in silico prediction (PASS and ADME) and molecular docking studies of key drugs were conducted.

## 2. Results and Discussion

### 2.1. Chemical Composition of Apium graveolens Oil (AGO) by GC–MS Analysis

**Figure 1:**
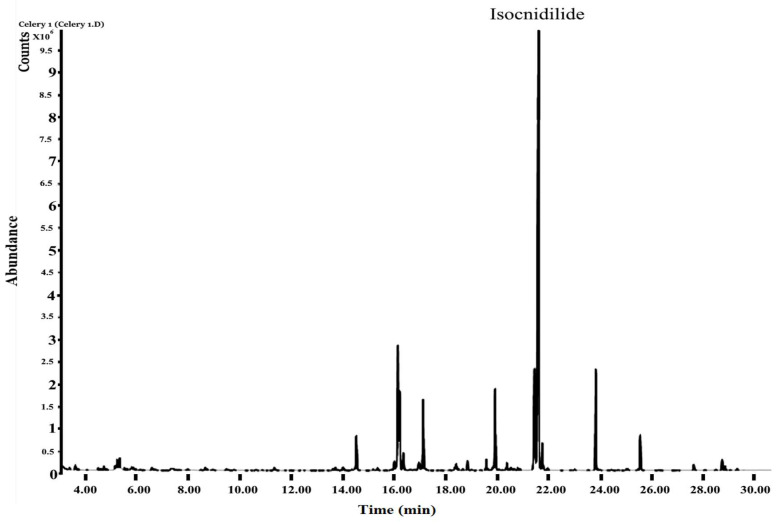
Chromatogram of *Apium graveolens* oil.

The *Apium graveolens* leaves produced 0.03% V/W of essential oil and clearly show low yield. Chemical composition of AGO was analyzed with the help of a developed GC–MS chromatogram (Figure 1). Table 1 presents the results of the GC–MS analysis of compounds. Benzofuranone, isocnidilide (40.1%) was found to be the predominant component of AGO, with other benzofuranone senkyunolide A (8.5%), 3-butylphthalide (5.36%), and Ligustilide (2.84%). The pharmacological active sesquiterpenes such as β-selinene (8.52%), dihydroagarofuran (5.21%), kessane (4.72%), and caryophyllene (2.42%) were found in significant amounts. Other components such as phytyl acetate (5.42%) and caryophyllene (2.42%) were also identified in significant amounts.

The composition of volatile compounds in AGO grown in the Saudi Arabia Al-Kharj region is different from that identified in the Tunisian cultivar, which exhibited 3-butylphthalide (38.4%) and 3-butyl-4,5-dihydrophthalide or senkyunolide A (34.2%) as the major components [1]. On the other hand, the present sample demonstrated isocnidilide or *cis*-neocnidilide (40.1%) as the major component, with 3-butylphthalide (5.36%) and senkyunolide A (8.48%) as minor components. The volatile compounds in the essential oil from South Korean celery exhibited different compositions from those in the essential oil from both Tunisian and Saudi cultivars [9]. Gold and Wilson (1963) also reported different concentrations of phthalide than those observed in the present cultivars [22]. Uhlig et al. (1987) reported sedanenolide as the main contributor of the characteristic odour of celery leaves [23]. In the present study, isocnidilide (sedanenolide; 40.1%) was identified as the major phthalide component, whose proportion was found to be higher than that reported in the Libyan and Korean cultivars [9,24,25]. Lund et al. (1973) stated that phthalide and β-selinene together contribute to the best quality of celery [26]. High concentrations of phthalides in the present sample make it a unique variety [24,25].

### 2.2. Antimicrobial Activity of Apium graveolens Oil

The antimicrobial activity of AGO was evaluated using the agar diffusion assay (Table 2). The strongest antimicrobial effect was observed at 40 mg/mL (4%) against the Gram-positive bacteria, *B. subtilis* (20.03 ± 0.06 mm) and *S. aureus* (18.6 ± 0.13 mm) and fungus *C. albicans* (20.13 ± 0.08 mm). The Gram-negative bacteria *E.coli* exhibited a comparatively smaller inhibition zone (11.46 ± 0.08 mm) at 40 mg/mL AGO. The MIC value of AGO against Gram-positive bacteria varied from 0.25 to 0.125%, whereas that for *C. albicans* was 0.125%, and that for Gram-negative bacteria was 0.5%. AGO was found to be less effective against Gram-negative bacteria (*E. coli* and *K. pneumoniae*).

However, relatively superior results were obtained for Gram-positive bacteria (*B. subtilis* and *S. aureus*) and the fungus, *C. albicans*. The most significant inhibition was exhibited by *B. subtilis* and *C. albicans,* with similar MIC values of 0.125%. However, the antioxidant potential of AGO in the present study is not consistent with the reported data. This discrepancy may be due to differences in the composition of essential oils [9]. *A. graveolens* extracts from Tunisia (mainly containing limonene and β-pinene) were reported to have strong inhibitory effects against *E. coli* and moderate inhibitory effect against *P. aeruginosa* and *S. aureus* [27]. However, the antibacterial potential of AGO in the present estimation against *E. coli* is not in support with the reported data. In the present study, AGO exhibited superior activity against *C. albicans*. This finding is concurrent with those of other reports [4,5].

### 2.3. Antioxidant and Anti-Inflammatory Activity of Apium graveolens Oil (AGO)

**Figure 2:**
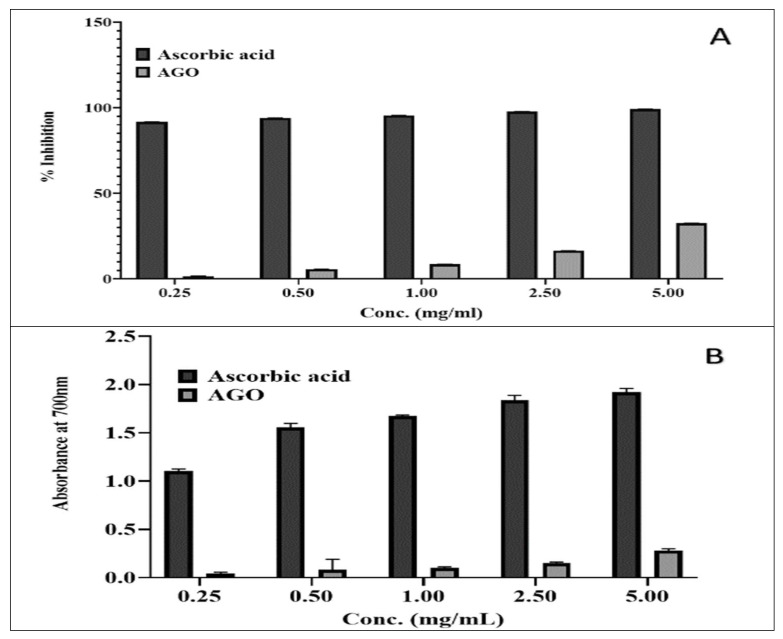
Antioxidant activity of the *Apium graveolens* oil (AGO), (**A**): DPPH; (**B**): FeCl_3_ method.

To examine the antioxidant activity, DPPH-induced FRS and ferric ion reducing assays were used, and the results of both assays are illustrated in Figure 2A,B, respectively. The percentage of DPPH-induced FRS capacity of AGO ranged from 1.580% ± 0.21% to 32.45% ± 0.2% at concentrations ranging from 0.25 mg/mL to 5 mg/mL. The range of absorption in ferric chloride increased from 0.043 ± 0.01 to 0.279 ± 0.02 as the concentration increased from 0.25 mg/mL to 5 mg/mL. The antioxidant power of the standard (ascorbic acid) was found to be the highest in both methods.

**Figure 3:**
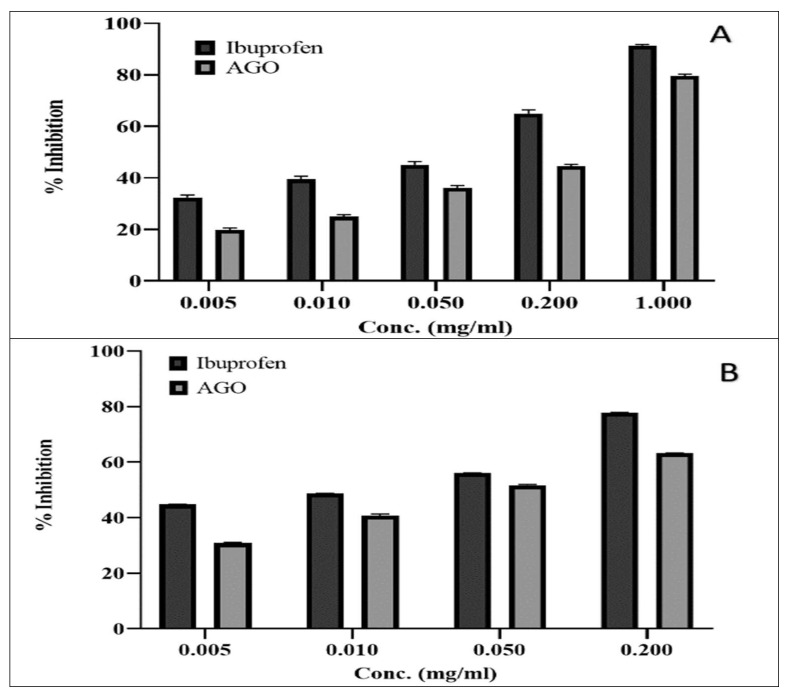
Anti-inflammatory activity of the *Apium graveolens* oil (AGO), (**A**): Egg albumin; (**B**): Trypsin inhibitory method.

Egg albumin- and trypsin-induced inflammation was evaluated for the anti-inflammatory activity assay; the results are illustrated in Figure 3A,B, respectively. The percentage inhibition of albumin-induced inflammation by AGO ranged from 19.8% ± 0.7% to 79.6% ± 0.7% at the concentration range of 0.05–1 mg/mL. The percentage inhibition of trypsin-induced inflammation increased from 30.8% ± 0.3% to 63.23% ± 0.1% as the concentration increased from 0.005 mg/mL to 0.2 mg/mL. The anti-inflammatory activity of AGO was found to be significantly higher (*p* < 0.001) than that of the standard (ibuprofen used as a positive control).

Ibuprofen, diclofenac, aspirin, and indomethacin are used as standard compounds for the evaluation of in vitro anti-inflammatory agents [28,29,30] in several studies. Ibuprofen has been reported as an NSAID and works by acting on prostaglandins cyclooxygenase-1 and 2 [31]. The present study revealed the superior anti-inflammatory potential of AGO, and the effects were found to be similar to those of ibuprofen. In another study, the in vivo anti-inflammatory effects of *A. graveolens* var. dulce (celery) leaves were found to be similar to those of indomethacin. This finding is concurrent with those of other studies [32,33]. The antioxidant activity of AGO at high concentrations (5 mg/mL) exhibited low DPPH scavenging activities and low absorption in ferric chloride method, which indicated the poor antioxidant properties of AGO. The anti-inflammatory activity of AGO at similar concentrations of standard (Ibuprofen) exhibited significant inhibition of albumin and trypsin-induced inflammation, which indicated the high anti-inflammatory properties of AGO. Several pharmacological studies have been conducted to determine the properties of celery essential oil. Pharmacological properties, such as anti-inflammatory, anticarcinogenic, insecticidal, antifungal, and mosquitocidal properties, of several phthalide compounds have been reported [4,34,35].

### 2.4. In Silico Molecular Docking, PASS, and ADME Prediction Studies

**Figure 4:**
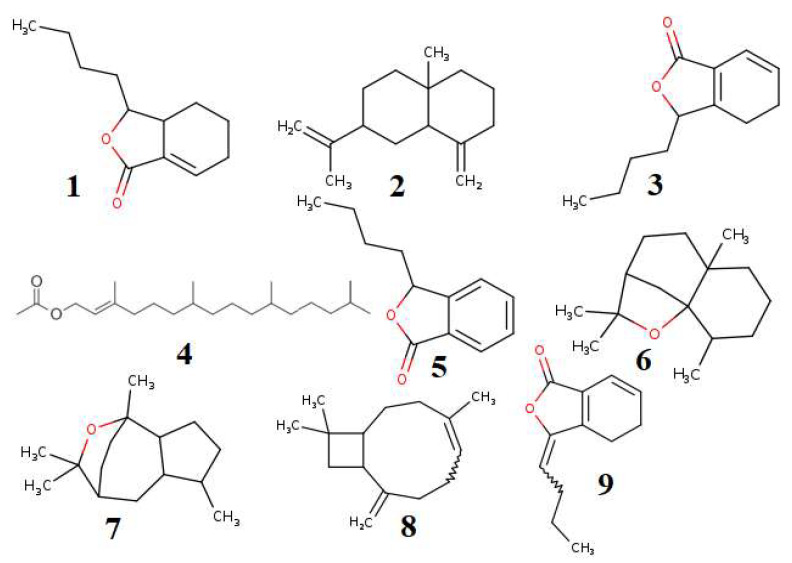
Major volatile compounds present in the AGO, Isocnidilide (**1**), β-Selinene (**2**), Senkyunolide (**3**), Phytyl acetate (**4**), 3-Butylphthalide (**5**), Dihydroagarofuran (**6**), Kessane (**7**), Caryophyllene (**8**), and Ligustilide (**9**).

Nine major volatile compounds present in the *A. graveolens* oil were selected for the activity prediction (Figure 4). The structure of the selected compound was drawn using ChemBioDraw [36] and converted to the SMILES format. Further, the SMILES format of the selected compounds was simulated in the SwissADME web tool [12]. The results of the expected activity using PASS tools [37] were presented in Table 3.

**Figure 5:**
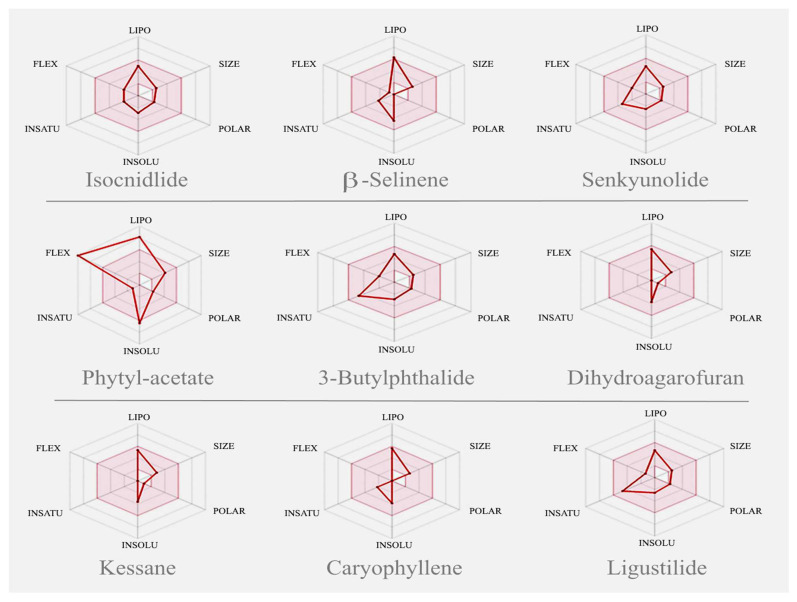
Bioavailability radar graph of nine compounds of AGO (pink area showed the drug likeness properties of the molecule).

**Figure 6:**
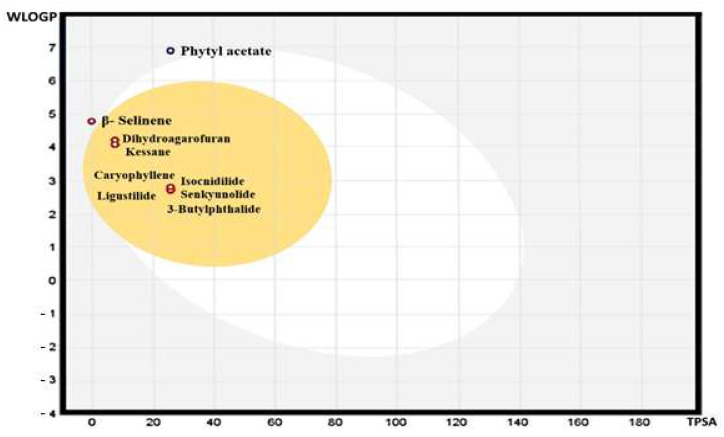
The graphical prediction (boiled-egg) of gastrointestinal absorption and brain penetration properties of selected compounds.

**Figure 7:**
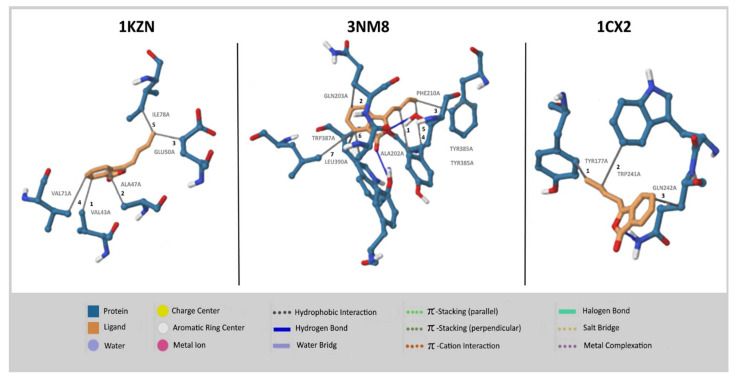
Interaction of 1kzn, 3NM8 and 1CX2 protein with isocnidilide inhibitor.

Results of Swiss ADME obtained from major compounds are reported in Table 3. The bioavailability radar graph, which represents physicochemical properties, pharmacokinetics, and drug-likeness properties of compounds present in AGO, was analyzed using SwissADME software [12]; the results are reported in Figure 5. The gastrointestinal absorption and brain penetration properties of selected compounds are illustrated in Figure 6. In silico molecular docking was performed only on isocinidilide against three receptors, namely DNA gyrase (1KZN), tyrosinase (3NM8), and cyclooxygenase-2 (1CX2), to identify the critical ligand–protein interactions, and the results are displayed in Figure 7. The results of binding energy and hydrophobic interactions are reported in Table 4.

The pharmacological activity spectra of nine major compounds were determined using PASS software [37]. Antioxidant activities of the AGO volatile compounds exhibited the lowest Pa (probability “to be active”) range (0.1–0.48). Among the identified compounds, phytyl acetate and isocnidilide were found to exhibit acceptable Pa values (0.48 and 0.46, respectively). However, other compounds were found to exhibit negligible antioxidant effects as per PASS prediction. The overall PASS prediction supported the present in vitro lower antioxidant potential of AGO. Antibacterial activities of the AGO volatile compounds exhibited a superior Pa range (0.29–0.44). Among the identified compounds, caryophyllene (0.44), senkyunolide A (0.42), phytyl acetate (0.41), and kessane (0.40) were found to exhibit good antibacterial effects. However, major compounds such as β-selinene (0.34) and isocnidilide (0.32) were predicted to have comparatively low antibacterial activities.

Overall, the PASS prediction supported the present in vitro antibacterial potential (MIC: 0.125–0.5%)) of AGO compounds. The Pa value of major compounds such as isocnidilide (0.5), β-selinene (0.53), senkyunolide A (0.51), caryophyllene (0.58), and phytyl acetate (0.61) for the antifungal potential is higher than that of these compounds for antibacterial effects. Hence, the PASS prediction supports the present high in vitro antifungal potential of AGO. The anti-inflammatory potential of the AGO volatile compounds exhibited the highest Pa range (0.27–0.76). Major compounds, namely isocnidilide (0.71), β-selinene (0.76), caryophyllene (0.74), and phytyl acetate (0.6), exhibited an excellent Pa value. The other predicted compounds also exhibited superior anti-inflammatory potential. Thus, the PASS prediction supports the present superior in vitro anti-inflammatory properties of AGO. The results obtained from in silico ADME Swiss software studies clearly indicated that all the selected volatile compounds have drug-like properties with no violation of any of the drug-likeness rules.

Outcomes of the predictor values of lipophilicity (total polar surface area (TPSA), solubility, and consensus log P_o/w_), pharmacokinetics and toxicity (gastrointestinal (GI) absorption, blood–brain barrier (BBB), P-gp substrate, CYP1A2 inhibitor, CYP2C19 inhibitor, CYP2C9 inhibitor, CYP2D6 inhibitor, and CYP3A4 inhibitor), and drug-likeness (Lipinski and bioavailability score) in these molecules were found to be in agreement with the crucial rules of drug-likeness. The TPSA of most of the compounds was 26.30 Å^2^, except for β-selinene, phytyl acetate, and caryophyllene, and these compounds exhibited a TPSA value of 0.00 Å^2^. Thus, the high GI absorption was predicted for most of the compounds, including that for the major compound isocnidilide. Thus, most compounds can be absorbed easily by the GI tract. The Swiss-prediction outcome also exhibited that all the compounds, except phytyl acetate, cannot be affected by the P-glycoprotein of the central nervous system (CNS). Drug-like properties and GI absorption of the volatile compounds present in AGO were also represented by the bioavailability radar graph and boiled-egg prediction. The pink area of the bioavailability radar graph represents drug-likeness, and the compounds present in the yellow zone, except β-selinene and phytyl acetate, can permeate through the blood–brain barrier (BBB).

The antimicrobial and anti-inflammatory studied have been proved by docking interaction isocnidilide with DNA gyrase, tyrosinase inhibitors, and COX-2 inhibitor. The docking studies suggested that −6.82, −6.59, and −8.37 kcal/mol showed good antimicrobial property of isocnidilide as compared to anti-inflammatory and antioxidant properties (Table 4). DNA gyrase with isocnidilide showed VAL (43A, 71A), ALA (47A), GLU (50A), and ILE (78A) residual interactions, followed by 3NM8 (tyrosinase inhibitors) protein, which showed ALA (202A), GLN (203A), PHE (210A), TYR (385A, 348A), TRP (387A), and LEU (390A), and TYR (177A), TRP (241A) and GLN (242A) in COX-2 inhibitors (1CX2). DNA gyrase (1KZN) is considered a potential target to inhibit bacterial growth, while the tyrosinase inhibitors (3NM8) have known potential for the development of novel antibacterial agents [38,39]. Similarly, protein COX-2 inhibitors (1CX2) are targeted for the development of novel anti-inflammatory agents in many recent studies [40]. In this study, we have proved by molecular docking that isocnidilide may be a good antimicrobial, anti-inflammatory compounds by the analysis of ligand recognition. On the basis of interction and its binding energy, isocnidilide was found to be the most potent inhibitor of the 1CX2 receptor, followed by 3NM8 and 1KZN. Theoretically, isocnidilide exhibited superior binding energy of −8.37 kcal.mol^−1^, which is in agreement with the observed moderate anti-inflammatory activity.

## 3. Materials and Methods

### 3.1. Extraction of Essential Oil

*A. graveolens* leaves were collected in January 2017 from Al-Kharj (central region), located in the Kingdom of Saudi Arabia (KSA) and deposited at the Prince Sattam bin Abdulaziz University (PSAU) herbarium, Department of Pharmacognosy, College of Pharmacy, Al-Kharj, KSA, with voucher specimen number PSAU-CPH 10-2017. The leaves were dried in shade and ground into powder. *A. graveolens* dried powder (200 gm) was hydro-distilled using Clevenger apparatus for 4 h. *A. graveolens* essential oil (AGO) was purified using the method described by Alam et al. [41].

### 3.2. Gas Chromatography–Mass Spectrometry Analysis

The gas chromatography–mass spectrometry (GC–MS) analysis of AGO was performed on an Agilent-5977B mass spectrometer (Agilent Technologies, Santa Clara, CA, USA) coupled with Gas chromatography 7890b (Agilent Technologies, Santa Clara, CA, USA) and a HP-5MS capillary column (30 m × 0.25 mm i.d., 0.25-μm coating). An aliquot of diluted oils (0.1 µL, 10%) was injected in a splitless mode, where the injector temperature was maintained at 280 °C, and the flow rate was maintained at 1 mL/min using carrier gas (helium: 99.999%). The column temperature was initially set at 40 °C and held for 2 min, after which it was changed at the rate of 5 °C/min to 70 °C and held for 5 min. Finally, the temperature was changed at a rate of 3 °C/min to 290 °C and held for 5 min (isothermally). The mass spectroscopy operating parameters were as follows: electron impact mode with ionization voltage: 70 eV; quadrupole temperature: 150 °C; ion source temperature: 180 °C; and scan/mass range: 30–600 amu with the rate of 0.32 s/scan. The essential oil components were documented by comparing standard retention time, the literature [42], and data of the GC–MS system MS library (NIST 2017: National Institute of Standards and Technology).

### 3.3. Antimicrobial Activity

Susceptibilities of 2 Gram-positive bacteria, 2 Gram-negative bacteria, and 1 fungus were assessed against AGO. The selected strains were *Staphylococcus aureus* ATCC 25923, *Bacillus subtilis* ATCC 11774, *Escherichia coli* ATCC 11229, *Klebsiella pneumoniae* NCTC 9633, and *Candida albicans* ATCC 10231. These bacterial strains were collected from the College of Pharmacy, PSAU, Al-Kharj.

The inhibitory effects of AGO were determined using the agar well diffusion assay [41]. In this method, 50 μL of bacterial inoculum was spread onto sterile Mueller Hinton (MH) agar plates. A sterile cork-borer was used to cut a 6 mm well from the agar. Subsequently, each well was filled with 50 μL of different concentration (1–4%) of the essential oil, prepared in 5% dimethyl sulfoxide. The plates were kept at room temperature for 30 min and then incubated at 37 °C for 24 h. Circular inhibition zones were calculated in millimetres. The minimum inhibitory concentration (MIC) was calculated using the microdilution method [43], and the test was performed on MH broth. Six serial dilutions of AGO (2%–0.0625%) were prepared, and a 100 μL aliquot was diluted in MH broth containing 10^6^ CFU/mL. The plates were incubated at 30 °C ± 5 °C/24 h for bacteria and at 25 °C ± 5 °C for *C. albicans*. The lowest AGO concentration that completely inhibited microbial growth was considered MIC.

### 3.4. In Vitro Antioxidant

#### 3.4.1. Inhibition Power

The measurement of the DPPH (2,2-diphenyl-1-picrylhydrazyl, Sigma-Aldrich, St. Louis, MO, USA) free radical scavenging ability of AGO was performed according to Yusufoglu et al. [44]. Experiments were executed in triplicate. Briefly, 10 mL methanol solution of DPPH (1 mmolL^−1^) and different concentrations (10–1000 μg/mL) of AGO and ascorbic acid (standard) were prepared. The reaction mixtures containing 100 μL of samples/standard and 1900 μL of DPPH were incubated in dark at 22 °C for 30 min. A control containing DPPH and methanol was prepared. The free radical inhibition was measured by reading the absorbance (Abs) at 517 nm against the control by using an ultraviolet–visible (UV–VIS) spectrophotometer. The percentage (%) of free radical scavenging (FRS) ability of AGO was measured using the following equation:

FRS (%) ability of AGO = [Abs (control) − Abs (sample)/Abs (control)] × 100.

#### 3.4.2. Reducing Power

The ferric reducing capacity of AGO was evaluated using the K_3_Fe(CN)_6_–FeCl_3_ (potassium ferricyanide–ferric chloride) method [44]. Briefly, a solution containing 10% (*w/v*) trichloroacetic acid (TCA), 1% potassium ferricyanide (K_3_Fe(CN)_6_), 0.2 M phosphate-buffer (pH 6.6), and 0.1% ferric chloride (FeCl_3_) was prepared. The reaction mixture containing 200 µL of different concentrations (10–1000 μg/mL) of the standard and AGO, 2.5 mL of phosphate-buffer, and 2.5 mL of K_3_Fe(CN)_6_ was incubated for 20 min at 50 °C. The reaction was terminated by adding 2.5 mL of TCA, and the upper clear layer was separated through centrifugation at 1000 rpm for 10 min. Finally, 2.5 mL of the upper layer was mixed with 2.5 mL of distilled water and 0.5 mL of FeCl_3,_ and the Abs was measured at 700 nm by using the Genesys 10s UV–VIS spectrophotometer (Thermo Scientific, Madison, WI, USA).

### 3.5. Anti-Inflammatory Activity

Phosphate-buffered saline (PBS, pH  =  6.8), different dilutions of AGO and ibuprofen (5–200 µg/mL in PBS), 1% egg albumin, 20 mM Tris-HCl buffer, 0.8% casein, and 70% perchloric acid were prepared. Anti-inflammatory properties were assayed using the egg albumin [45] and trypsin [46] methods, with slight modifications.

#### 3.5.1. Albumin Denaturation Method

A reaction mixture containing 100 µL of sample/standard, 1000 µL of albumin solution, and 1400 µL of PBS was incubated for 15 min (at 37 °C) and then kept for 5 min (at 72 °C). This mixture was then cooled, and the Abs was determined at 660 nm by using a spectrophotometer. Each experiment was repeated thrice, and the percentage inhibition (protein denaturation) was determined as the mean ± standard deviation by using the following equation:

Denaturation (%) = [(1 − Abs (sample)/Abs (control) × 100]

#### 3.5.2. Proteinase Inhibitory Assay

For the trypsin assay, the reaction mixture containing 1000 µL of sample or standard, 0.06 mg trypsin, and 1 mL tris-HCl was incubated for 15 min (at 37 °C). Then, 1 mL of casein was added to the mixture, and the reaction mixture was incubated further for 20 min (at 37 °C). Then, 2 mL perchloric acid was added to the reaction mixture, and the mixture was centrifuged at 3000× *g* for 5 min. The reaction mixture containing all reagents except standard and samples was used as control. The Abs of the supernatant was measured at 210 nm. Each experiment was repeated thrice, and the percentage inhibition was determined as the mean ± standard deviation by using the following equation:

Inhibition (%) = [(1 − Abs (sample)/Abs (control) × 100]

### 3.6. In Silico Docking Studies

The structure of the selected compound was drawn using ChemBioDraw [36] for the prediction of activity spectra for substances (PASS) and pharmacokinetics (absorption, distribution, metabolism, and excretion (ADME)) studies. The legends were converted into the SMILES format. PASS is an online web tool [45] that predicts probable activity (Pa) and probable inactivity (Pi) and is applied to ‘drug-like’ substances [47]. The pharmacokinetic properties were also estimated using SwissADME software [12], which can predict the physiochemical properties, lipophilicity, drug-likeness, and toxicity.

The isocnidilide compound was selected for the docking study based on the higher percentage contents. The structures of antibacterial target protein DNA gyrase, PDB: 1KZN [48], antioxidant tyrosinase enzyme, PDB ID: 3NM8 [49], and anti-inflammatory target protein cyclooxygenase-2, PDB ID: 1CX2 [50] enzymes were revealed from the protein data bank.

DNA gyrase (PDB ID: 1KZN), tyrosinase (PDB ID: 3NM8), and cyclooxygenase-2 (PDB ID: 1CX2) protein have been selected for docking studies by AutoDock Tools 1.5.6, a protein-ligand docking tool [51]. At the beginning of docking, Kollman charges and Gasteiger charges were added to the protein and ligands, respectively. The grid was centered on the basis of target active site prediction within that form the binding sites as reported by Jin et al. [52]. The size of the grid box was 40 Å, which was the same for x, y, and z, with the grid center set to −9.554, 12.075, and 70.876 for x, y, and z, respectively, with spacing 0.5 Å. AutoGrid (Scripps Research, La Jolla, CA, USA) and AutoDock 4.0 programs were used to generate grid maps with 1000 time sensitivity. The best ten conformers were generated using Lamarckian Genetic Algorithm (4.2). The binding energy and inhibition constant for each pose was calculated, and the best selected poses were visualized using the protein-ligand profiler [53].

### 3.7. Statistical Analysis

All the data were statistically evaluated using GraphPad Prism software (Version 9.3.0, San Diego, CA, USA). The results are articulated as either average or mean  ±  SD of the triplicate experiments.

## 4. Conclusions

In vitro studies on AGO indicated the weak antioxidant activity, superior antimicrobial activity, and excellent anti-inflammatory activity of AGO. The compounds present in the AGO exhibited promising in silico results, where the PASS prediction supports the excellent anti-inflammatory activity, moderate antifungal activity, superior antibacterial activity, and low antioxidant activity, similar to the in vitro results. ADME Swiss software studies clearly indicated that the compounds present in the AGO have drug-like properties such as low CNS toxicity, and most of the compounds can be easily absorbed through the GI tract and can permeate through the BBB. The molecular docking results of isocnidilide, a major compound in AGO, on 1KZN, 3NM8, and 1CX2 theoretically exhibited agreement with the in vitro activities, as indicated by their significant protein–ligand interaction energy.

## Figures and Tables

**Table 1:** Chemical composition of *Apium graveolens* oil (AGO).

S.N.	Metabolites	RT (min)	Content (%)
1	Oxime-, methoxy-phenyl-_	3.14	0.25
2	*o*-Anisidine, *N*-trimethylsilyl-	3.61	0.24
3	1,5-Dimethoxy-1,3,5-trimethyltrisiloxane	4.72	0.17
4	Benzene, 1-methyl-3-(1-methylethyl)-	5.15	0.14
5	d-Limonene	5.23	0.37
6	*trans*-β-Ocimene	5.33	0.43
7	γ-Terpinene	5.80	0.13
8	1-Propanol, 2,2-dimethyl-, benzoate	6.58	0.29
9	2,4,6-Octatriene, 2,6-dimethyl-, (*E*,*E*)-	7.36	0.4
10	*p*-Menth-1-en-4-ol	8.49	0.1
11	Naphthalene	8.65	0.22
12	*trans*-Carveol	9.46	0.12
13	3-Heptyne-2,5-diol, 6-methyl-5-(1-methylethyl)-	11.33	0.33
14	β-Damascenone	13.61	0.11
15	Isopinocarveol	13.71	0.24
16	Diphenyl ether	14.01	0.28
17	Caryophyllene	14.52	2.42
18	Humulene	15.34	0.2
19	α-Curcumene	15.99	0.55
20	β-Selinene	16.14	8.52
21	Dihydroagarofuran	16.20	5.21
22	α-Selinene	16.35	1.34
23	7-Octen-4-one, 2,6-dimethyl-	16.95	0.76
24	Kessane	17.11	4.72
25	Caryophyllene oxide	18.40	0.65
26	1-Undecanol	18.48	0.11
27	Hexadecane	18.66	0.1
28	2-Cyclopenten-1-one, 2,3,4,5-tetramethyl-	18.84	0.61
29	Hexahydro-3-butylphthalide	19.57	0.69
30	Perilla alcohol angelate	19.83	0.19
31	3-butylphthalide	19.92	5.36
32	1(3H)-Isobenzofuranone, 3-butylidene-	20.36	0.49
33	*N*,*N*’-Diacetyl-1,4-phenylenediamine	20.53	0.19
34	(3-Methylphenyl) methanol, 2-methylbutyl ether	20.79	0.17
35	Isocnidilide	21.60	40.1
36	Senkyunolide	21.44	8.48
37	*trans*-Ligustilide	21.74	2.84
38	*trans*-Sedanolide	21.95	0.15
39	Phytyl acetate	23.82	5.42
40	9,12,15-Octadecatrien-1-ol, (*Z*,*Z*,*Z*)-	25.02	0.1
41	Hexadecanoic acid, methyl ester	25.54	1.12
42	Falcarinol	27.63	0.40
43	9,11-Octadecadienoic acid, methyl ester, (*E*,*E*)-	28.73	0.66
44	Linolenic acid, methyl ester	28.84	0.29
45	Methyl stearate	29.31	0.12
Total Percentage Area			95.78%

**Table 2:** Antimicrobial activities of *Apium graveolens* oil (AGO).

Organisms Tested	Zone of Inhibition (In Millimeter)			MIC * (% *V*/*V*)
	1%	2%	4%	
***S. aureus-ATCC26923***	13.4 ± 0.02	15.4 ± 0.01	18.6 ± 0.13	0.25
***B. subtilis-ATCC11774***	15.13 ± 0.08	17.3 ± 0.08	20.03 ± 0.06	0.125
***E.coli-ATCC11229***	6.63 ± 0.04	8.05 ± 0.08	11.46 ± 0.08	0.5
***K. pneumoniae-NCTC9633***	9.5 ± 0.09	11.13 ± 0.08	14.47 ± 0.02	0.5
***C. albicans-ATCC10231***	15.53 ± 0.09	18.5 ± 0.09	20.13 ± 0.08	0.125

* MIC (Minimum inhibitory concentration).

**Table 3:** In silico ADMET profile of AGO compounds.

Entry	Isocnidilide	β-Selinene	Senkyunolide	Phytyl Acetate	3-Butylphthalide	Dihydroagarofuran	Kessane	Caryophyllene	Ligustilide
Rt	21.60	16.14	21.44	23.82	19.92	16.21	17.12	14.52	21.74
Area (82%)	40.1	8.5	8.5	5.4	5.4	5.1	4.7	2.4	1.9
Mol wt g/mol	194.27	204.35	192.25	338.57	190.24	222.37	222.37	204.35	190.24
TPSA*	26.30 Å^2^	0.00 Å^2^	26.30 Å^2^	26.30 Å^2^	26.30 Å^2^	9.23 Å^2^	9.23 Å^2^	0.00 Å^2^	26.30 Å^2^
Consensus * Log P_o/w_	2.87	4.50	2.71	6.67	2.81	3.80	3.68	4.24	2.75
Water Solubility *	Soluble	Soluble	Soluble	Poorly soluble	Soluble	Soluble	Soluble	Soluble	Soluble
GI absorption **	High	Low	High	low	High	High	High	Low	High
BBB permeant **	Yes	no	Yes	no	yes	yes	yes	no	yes
P-gp substrate **	no	no	no	yes	no	no	no	no	no
CYP1A2 inhibitor **	no	no	no	no	yes	no	no	no	yes
CYP2C19 inhibitor **	no	Yes	no	no	no	no	no	yes	no
CYP2C9 inhibitor **	Yes	Yes	no	yes	no	yes	no	yes	no
CYP2D6 inhibitor **	no	no	no	no	no	no	no	no	no
CYP3A4 inhibitor **	no	no	no	no	no	no	no	no	no
Lipinski ***	yes	yes	yes	yes	yes	yes	yes	yes	yes
Bioavailability Score ***	0.55	0.55	0.55	0.55	0.55	0.55	0.55	0.55	0.55
PASS (Pa > Pi)									
Anti-inflammatory	0.71 > 0.01	0.76 > 0.01	0.42 > 0.08	0.6 > 0.03	0.49 > 0.06	0.3 > 0.15	0.27 > 0.12	0.74 > 0.01	0.38 > 0.02
Antibacterial	0.32 > 0.05	0.34 > 0.04	0.42 > 0.02	0.42 > 0.03	0.39 > 0.03	0.29 > 0.06	0.4 > 0.03	0.44 > 0.02	0.3 > 0.06
Antifungal	0.5 > 0.03	0.53 > 0.02	0.51 > 0.02	0.61 > 0.01	0.42 > 0.06	0.31 > 0.08	0.33 > 0.07	0.58 > 0.02	0.29 > 0.08
Antioxidant	0.46 > 0.06	0.12 > 0.12	0.22 > 0.04	0.48 > 0.01	0.20 > 0.05	-	0.13 > 0.12	0.17 > 0.07	0.14 > 0.10

Lipophilicity *, Pharmacokinetics and toxicity **, Drug Likeness ***.

**Table 4:** The binding energy and Hydrophobic Interactions for isocnidilide drugs with target proteins.

Protein (PDB)	Binding Energy	Residue	AA	Distance	Ligand Atom	Protein Atom
1KZN	−6.82 kcal/mol	43A	VAL	3.28	1787	271
		47A	ALA	3.70	1783	306
		50A	GLU	3.53	1792	331
		71A	VAL	3.51	1786	517
		78A	ILE	3.87	1792	582
3NM8	−6.59 kcal/mol	202A	ALA	3.73	5452	1671
		203A	GLN	3.88	5450	1677
		210A	PHE	3.51	5457	1763
		385A	TYR	3.74	5455	3526
		348A	TYR	3.83	5457	3524
		387A	TRP	3.52	5452	3551
		390A	LEU	3.63	5452	3589
1CX2	−8.37 kcal/mol	177A	TYR	3.06	2904	1732
		241A	TRP	3.13	2903	2392
		242A	GLN	3.76	2895	2399

## Data Availability

The data presented in this study are available in this article.

## Sample Availability

Samples of the plants are available from the authors.
